# A new record of Avrainvillea cf. erecta (Berkeley) A. Gepp & E. S. Gepp (Bryopsidales, Chlorophyta) from urbanized estuaries in the Hawaiian Islands

**DOI:** 10.3897/BDJ.6.e21617

**Published:** 2018-10-05

**Authors:** Rachael M Wade, Heather L Spalding, Kimberly A Peyton, Kevin Foster, Thomas Sauvage, Matthew Ross, Alison R Sherwood

**Affiliations:** 1 University of Hawai‘i at Mānoa, Honolulu, United States of America University of Hawai‘i at Mānoa Honolulu United States of America; 2 Division of Aquatic Resources, Honolulu, United States of America Division of Aquatic Resources Honolulu United States of America; 3 U.S. Fish and Wildlife Service, Honolulu, United States of America U.S. Fish and Wildlife Service Honolulu United States of America; 4 Smithsonian Marine Station, Fort Pierce, United States of America Smithsonian Marine Station Fort Pierce United States of America; 5 Unaffiliated, Honolulu, United States of America Unaffiliated Honolulu United States of America

**Keywords:** *
Avrainvillea
*, Bryopsidales, Chlorophyta, estuary, Hawai‘i, invasive, *rbc*L, seagrass, *tuf*A

## Abstract

**Background:**

A second species in the siphonous green algal genus *Avrainvillea* was recently discovered off the island of O‘ahu in the Main Hawaiian Islands. Specimens were collected from Honolulu Harbor, including its entrance channel, and near Ke‘ehi Harbor. These locations are both in Mālama Bay on O‘ahu’s south shore in or adjacent to urbanized estuaries, respectively. *In situ* observations, morphological and molecular assessments were conducted to examine the alga’s habit and distribution, as well as to assess its putative species identification.

**New information:**

The alga occurred in sand as single individuals or in clusters of several individuals at both sites, and near or within seagrass beds (*Halophila decipiens*) and algal meadows composed of the green alga *Halimeda kanaloana* and an unidentified *Udotea* species at the Ke‘ehi Harbor site. All analyses supported both populations as representative of the same taxa, reported until further investigation in the broad Pacific as Avrainvillea cf. erecta based on morphological and molecular analyses. This record of a second *Avrainvillea* species in Hawai'i is of particular concern considering that an alga recognized as *A. amadelpha*, first observed in 1981 from two locales on O‘ahu’s south shore, has become invasive in Hawai‘i’s intertidal to mesophotic environments.

## Introduction

The siphonous green algal order Bryopsidales includes over 500 extant species (Guiry and Guiry 2017). This diversity is largely due to the evolution of unique traits, which allows them to become significant and persistent members of the marine environment. These characters include macroscopic unicellularity (Vroom and Smith 2001), rapid growth (Smith et al. 2004), adaptability to low nutrient environments (Lobban and Harrison 1994, Smith et al. 2004, Malta et al. 2005), chemical defenses (unpalatability and subsequent escape from predators, e.g. Hay et al. 1987, Becerro et al. 2001, Baumgartner et al. 2009), and vegetative reproductive ability via fragmentation (Hillis-Colinvaux et al. 1965, Walters and Smith 1994, Vroom et al. 2003, Wright and Davis 2006). The ecological success of some members of the Bryopsidales when introduced to new environments has been strongly demonstrated by the invasion and persistence of *Caulerpa taxifolia* (M. Vahl) C. Agardh in the Mediterranean (Meinesz et al. 2001) and Codium fragile ssp. tomentosoides (van Goor) P. C. Silva (= ssp. fragile (Suringar) Hariot) across the globe (Provan et al. 2004).

An unknown species of *Avrainvillea* was first documented on the western shore of O‘ahu in 1981. By 1985, the alga had spread to the inter- and subtidal environments of O‘ahu’s south shores. This distribution was documented by Brostoff (1989), who identified the alga as *Avrainvillea amadelpha* (Montagne) A. Gepp & E. S. Gepp. However, Brostoff 1989 stated that the alga also closely resembled three other species; more recent morphological and molecular analyses have also not been able to conclusively identify the alga to the species level because of its morphological plasticity, but *A. amadelpha* is most likely incorrect (Wade et al. 2015). Thus, throughout the manuscript this species will be referred to provisionally as “*A. amadelpha*”. Interestingly, an invasive alga identified as "*A. amadelpha*" was recently recorded in the Mediterranean as well (Verlaque et al. 2017).

**Figure 1.**
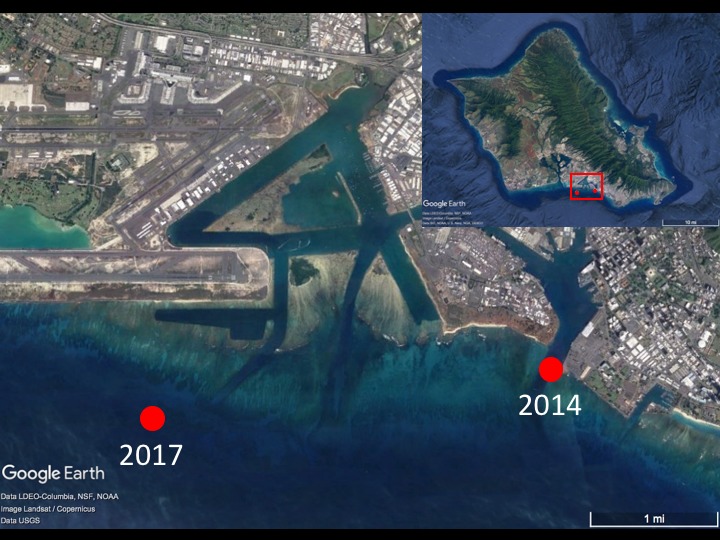
Avrainvillea cf. erecta occurrence map. Specimens were collected from the Honolulu Harbor entrance channel in 2014 during a regular seagrass survey by the U.S. Fish and Wildlife Service and the Hawai‘i Department of Land and Natural Resources, Division of Aquatic Resources and again near Ke‘ehi Harbor in 2017 during a non-research dive by M. Ross.

More recently, a population of a second *Avrainvillea* species, distinct in habit from "*A. amadelpha*", was discovered on October 14-16, 2014 in Honolulu Harbor, including its entrance channel and turning basin, from 12-15 m depths (Fig. 1). Honolulu Harbor is the principal seaport for all of the Hawaiian Islands, handling approximately 80% of goods imported into the islands and servicing both international and domestic vessels. The alga was found again on April 22, 2017 seaward of Ke‘ehi Lagoon from 25-40 m depths; this area is near the Ke‘ehi Boat Harbor in the vicinity of offshore anchorages for large commercial vessels, as well as an urbanized and commercialized area of Honolulu. Both sites are located in Mālama Bay on the south shore of O‘ahu in the Main Hawaiian Islands (Fig. 1). Here we provide *in situ* observations of these populations in urbanized estuaries, and assess the molecular identity of the new species record and its morphology in comparison to the previously reported "*A. amadelpha*."

## Materials and methods

### In situ observations

A quantitative seagrass community survey using SCUBA from 12-18m depth was conducted jointly by the U.S. Fish and Wildlife Service and State of Hawai‘i Department of Land and Natural Resources - Division of Aquatic Resources in Honolulu Harbor from October 14-16, 2014 as part of regular benthic surveys in preparation of scheduled dredging. Field data were collected in the planned dredge footprint at eight locations within the turning basin and entrance channel, locations which are referred to as "Impact Sites" (Suppl. material 1). An additional eight sites were sampled outside of the dredging areas, referred to as "Control Sites." Five-minute swims were made at each site to record other benthic species, during which time a population of *Avrainvillea* was discovered. After this unexpected discovery, rapid assessments and collections of this alga were undertaken at each site where it was observed.

Specimens were also discovered on April 22, 2017 offshore of Ke‘ehi Lagoon, south O‘ahu using SCUBA at 25-40 m depths. Subsequent qualitative SCUBA surveys were conducted at 20-30 m depths at three sites near the original collection site on May 18, 2017 to make qualitative observations regarding its habitat and associated organisms.

### Morphological characterization

Two specimens collected in 2014 (BISH768338-9) and six collected in 2017 (BISH768278-83) that included what appeared to be mature and juvenile forms or possibly ecotypes were selected for morphological and molecular characterization (Suppl. material 2). Additional specimens provided by co-authors and collaborators were also assessed for species identification in the same manner (Suppl. material 2). Morphology was evaluated using 12 macroscopic characters and 21 microscopic characters (Suppl. material 3). Tentative species-level identification was determined by comparison with original species descriptions (Decaisne 1842, Agardh 1887, Gepp and Gepp 1908, Gepp and Gepp 1911, Olsen-Stojkovich 1985, Littler and Littler 1992) and the re-evaluated descriptions and dichotomous keys provided by Olsen-Stojkovich (1985) and Littler and Littler (1992). In particular, the groups described by Olsen-Stojkovich (1985) that are a result of similarity-graph clustering using morphological characters and growth habit (i.e. the “longicaulis”, “nigricans”, and “obscura” groups) were used to compare the newly recorded *Avrainvillea* sp. and the previously recorded “*A. amadelpha*”.

### Molecular assessment

In addition to the specimens used for morphological assessment, two type specimens of heterotypic synonyms of *Avrainvillea erecta* (*Chloroplegma papuanum* Zanardini and *Rhipilia andersonii* G. Murray) were borrowed from the Natural History Museum of London and included in our molecular assessment (morphological assessment, and therefore additional destructive sampling, was not permitted). DNA extraction was completed using the OMEGA E.Z.N.A^®^ Plant DNA Kit (OMEGA bio-tek, Norcross, GA U.SA.). For the two type specimens, the protocol developed by Hughey et al. (2001) was used. DNA extracts were amplified for portions of two chloroplast gene regions: the 5' end of *rbc*L (ribulose-1,5-bisphosphate carboxylase/oxygenase – large subunit, 562 bp) and *tuf*A (elongation factor Tu, 714 bp). These gene regions were selected as informative and reliably sequenced regions for siphonous green algae (Leliaert et al. 2014) and their use in previous Bryopsidales phylogenetic studies (e.g. Curtis et al. 2008, Verbruggen et al. 2009a, Verbruggen et al. 2009b, Wade and Sherwood 2017, Wade and Sherwood 2018). For the type specimens, a modified protocol was used with short, overlapping fragments for each gene, rather than amplifying the entire fragment at once; a new protocol was developed for *rbc*L and the *tuf*A protocol described by Sauvage et al. 2014 was used. For *rbc*L, three fragments were amplified using the forward (rbcLF) and reverse (rbcLR) primers developed by Pierce et al. (2006) and newly developed internal primers rbcL223R (5' KTCTTCACCDGCDACTGGTT 3'), 204F(5' GAACCAGTHGCHGGTGAAGA 3'), 400R(5' GWGGHCCTTGRAAHGTTTT 3'), and 381F(5' ACRTTTCAAGGVCCACCACA 3'). Sequences were edited and aligned with previously generated sequences and reference sequences available on GenBank using Geneious 7.1.8 (BioMatters, Auckland, N.Z.). The two gene alignments were then concatenated for phylogenetic analyses. Model selection (AICc and BIC: GTR+I) and partition scheme (no partitions) were determined using PartitionFinder 1.1.0 (Lanfear et al. 2012). Maximum likelihood phylogenetic reconstruction was conducted using RAxML-HPC2 on XSEDE 8.1.11 (Stamatakis 2014) for 1,000 bootstrap generations. Bayesian inference was conducted using MrBayes on XSEDE 3.2.2 (Huelsenbeck and Ronquist 2001, Ronquist and Huelsenbeck 2003) for 2 x 10^6^ generations with chain sampling every 1,000 generations and a burnin value of 25% until congruence was met (standard deviation of split frequencies <0.05). Both RAxML and MrBayes were accessed on the CIPRES Science Gateway (Miller et al. 2010).

## Taxon treatments

### Avrainvillea erecta

Gepp & Gepp 1911

#### Materials

- **Type status:** Other material. **Occurrence:** catalogNumber: BISH768278-83; recordedBy: Matthew Ross; individualCount: 6; otherCatalogNumbers: ARS09414,-09417,-09418, -09429,-09431,-09432; associatedSequences: MF872080-85, MF872105-110; **Taxon:** scientificName: Avrainvillea cf. erecta; kingdom: Plantae; phylum: Chlorophyta; class: Ulvophyceae; order: Bryopsidales; family: Dichotomosiphonaceae; genus: Avrainvillea; specificEpithet: erecta; scientificNameAuthorship: (Berkeley) A. Gepp & E.S. Gepp; **Location:** country: USA; municipality: Honolulu; locality: Mālama Bay, seaward of Ke‘ehi Lagoon; minimumDepthInMeters: 25; maximumDepthInMeters: 40; decimalLatitude: 21.29; decimalLongitude: 157.9205; georeferenceProtocol: GPS; **Identification:** identifiedBy: Rachael M. Wade; dateIdentified: May-2017; identificationReferences: Olsen-Stojkovich 1985; **Event:** eventDate: Apr-22-2017; **Record Level:** language: en; basisOfRecord: PreservedSpecimen
- **Type status:** Other material. **Occurrence:** catalogNumber: BISH768338-9; recordedBy: Kimberly Peyton, Kevin Foster, Paul Murakawa; individualCount: 2; otherCatalogNumbers: ARS09436-7; associatedSequences: MF969093-6; **Taxon:** scientificName: Avrainvillea cf. erecta; kingdom: Plantae; phylum: Chlorophyta; class: Ulvophyceae; order: Bryopsidales; family: Dichotomosiphonaceae; genus: Avrainvillea; specificEpithet: erecta; scientificNameAuthorship: (Berkeley) A. Gepp & E.S. Gepp; **Location:** country: USA; municipality: Honolulu; locality: Mālama Bay, Honolulu Harbor; minimumDepthInMeters: 12; maximumDepthInMeters: 15; decimalLatitude: 21.30; decimalLongitude: 157.8689; georeferenceProtocol: GPS; **Identification:** identifiedBy: Rachael M. Wade; dateIdentified: Aug-2017; identificationReferences: Olsen-Stojkovich 1985; **Event:** eventDate: Oct-15-2015; **Record Level:** language: en; basisOfRecord: PreservedSpecimen
- **Type status:** Other material. **Occurrence:** associatedSequences: MH938452; **Taxon:** scientificName: Avrainvillea erecta; acceptedNameUsage: Avrainvillea erecta Gepp & Gepp 1911; originalNameUsage: Chloroplegma papuanum Zanaradini 1878; kingdom: Plantae; phylum: Chlorophyta; class: Ulvophyceae; order: Bryopsidales; family: Dichotomosiphonaceae; genus: Avrainvillea; specificEpithet: erecta; taxonomicStatus: heterotypic synonym; **Location:** waterBody: Pacific Ocean; country: Indonesia; stateProvince: Papua; **Identification:** identifiedBy: Zanardini; dateIdentified: 1878; **Event:** year: 1872; month: May; fieldNotes: Collected by Odoardo Beccari; **Record Level:** institutionID: BM000561613; basisOfRecord: PreservedSpecimen

#### Description

##### In situ observations

During the 2014 seagrass community survey, the newly discovered *Avrainvillea* sp. was observed at six of 16 survey sites in the Honolulu Harbor entrance channel from 12-15m depths (four "Control Sites", two "Impact Sites"; Suppl. material 1). The two morphologies (blade-like versus assemblage of loose siphons) differed in their exposure to water flow – individuals with a completely formed blade were often elevated and fully exposed to water motion, while individuals with a loose assemblage of siphons were in depressions or divots and therefore protected (K. Peyton, unpublished data). This water motion effect was also supported by informal experimentation: in water tables without water flow, blades were observed to unweave and become loose assemblages while specimens with water flow maintained the blade morphology (K. Peyton, unpublished data).

In 2017, the newly recorded *Avrainvillea* sp. was observed as single individuals or in patches with 10-20 individuals per m^2^ (estimated visually) in areas with deep sand (Fig. 2a, b). The dominant vegetation in these sand beds was the seagrass *Halophila decipiens* Ostenfeld, patches of the macroalga *Halimeda kanaloana* Vroom, and an unidentified *Udotea* sp. Several individuals were observed with feeding scars (large bite marks), giving some thalli a U-shaped appearance. The holdfasts of larger, more mature individuals protruded from the sediment by approximately 1-5 cm, creating a conical mound at the base of the alga. Individuals were generally clean and not heavily epiphytized. The two morphologies at this location experienced very little water motion due to attenuation of wave motion with depth, and therefore were most likely the result of differences in age. The individuals with spherical assemblages of loose siphons were consistently much smaller in thallus size than the well-formed blade morphology.

##### Morphological characterization

The specimens were olive-green upon collection and dried to a darker green with fulvous, or tawny, coloration (Fig. 2c, d). Specimens were categorized as mature adults (BISH768278), immature adults (BISH768279-80) or juveniles (BISH768281-3). Adult individuals, both mature and immature, ranged in overall length from 6.7-15.8 cm; frond length ranged from 3.6-10.8 cm. Each adult thallus was distinctly differentiated into a holdfast, stipe, and blade. The rhizomatous holdfasts comprised up to 46% of the total thallus length. The thin stipes of adult individuals supported a lightly zonate and reniform to sub-reniform blade; margins appeared to be composed of loose aggregates of siphons, but not necessarily lacerate. Individuals that appeared to be juveniles consisted only of a holdfast and a spherical assemblage of loose siphons, which appeared to be in the beginning stages of forming a blade. Siphons throughout the specimens (e.g. margin, blade, stipe, and holdfast) were mostly cylindrical to slightly torulose and measured in width 11.1-(25.4-59.1)-93.1 µm with acute and deep constrictions above the dichotomies (Fig. 2e). Constrictions were also common below the dichotomy, except in the holdfast siphons. Apices were primarily rounded, but also rarely blunt or sub-clavate. Siphons appeared olive green, transparent, or fulvus, which was attributed to overall siphon color and/or chloroplast pigmentation. These characters and measurements suggest affinity with the description of *Avrainvillea erecta* (Berkeley) A. Gepp & E.S. Gepp and their further morphological characterization by Olsen-Stojkovich (1985).

##### Molecular assessment

**Figure 3.**
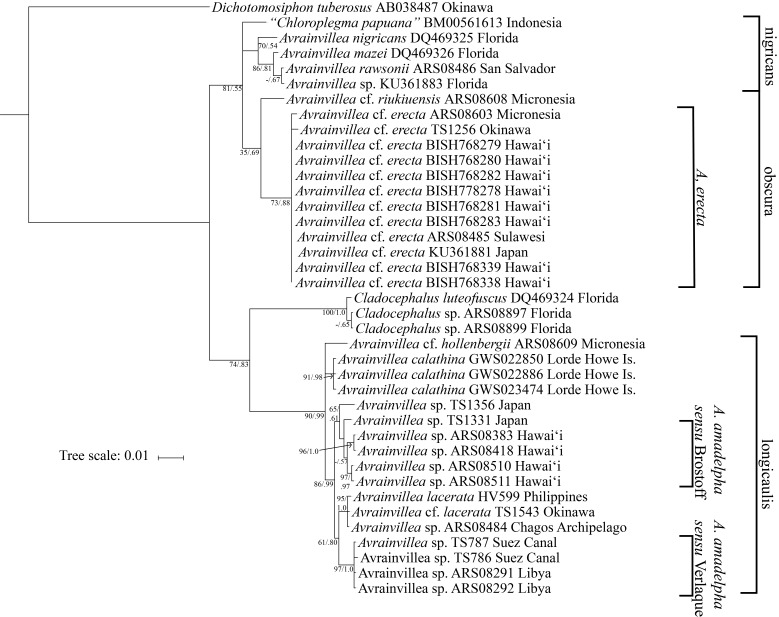
Bayesian inferred phylogeny from the *rbc*L and *tuf*A concatenated alignment. The leftmost clade notations specify the two *Avrainvillea* spp. found in Hawai‘i, the righmost clade notations specify the *Avrainvillea* groups described by Olsen-Stojkovich 1985. Scale bar = substitutions per site. Nodal support values represent Maximum Likelihood bootstrap values and Bayesian inference posterior probabilities, respectively.

The majority of examined specimens were sequenced for both *rbc*L and *tuf*A, however, molecular characterization of historical material was only successful for *rbc*L for one of the heterotypic synonym type specimens - *Chloroplegma papuanum* BM000561613. The concatenated alignment of the two gene regions yielded a dataset of 1,360 bp. Both the Maximum Likelihood and Bayesian inference phylogenetic reconstructions strongly supported that the newly recorded *Avrainvillea* species, A. cf. erecta, was clearly distinct from Hawai‘i specimens identified as “*A. amadelpha*” (Brostoff 1989); these newly sequenced specimens belong to the “obscura” group while “*A. amadelpha*” clusters within the “longicaulis” group (Olsen-Stojkovich 1985) (Fig. 3). These analyses also support the monophyletic grouping of sequences from the newly sequenced specimens and those morphologically identified as A. cf. erecta (Berkeley) A. Gepp & E. S. Gepp from Japan and Micronesia. Although they exhibited two different morphs (loose siphons or blade), all specimens from the two Hawai‘i populations had identical DNA sequences.

## Discussion

The morphological and molecular characterization of the newly recorded *Avrainvillea* species showed most affinities to the description of *A. erecta* based on stipe length, blade habit, siphon width and morphology (constriction at dichotomy); however considering that we could not obtain material from type locality or the basionym type specimen (*Dichonema erectum* Berkeley 1842), we temporarily consider the newly recorded species as A. cf. erecta until further research can be conducted (Suppl. material 2, Fig. 3). For instance, the specimens closely resemble *A. obscura* (C.Agardh) J.Agardh, in part due to the description of the species’ ecomorphs that resemble both morphologies described here (Agardh 1887). However, a reduced stipe undifferentiated from a cuneate blade, general lack of blade zonation, and non-fulvous siphons of *A. obscura* make it a less likely match than *A. erecta.* The phylogenetic separation of the Hawai‘i specimens and the *Chloroplegma papuanum* specimen does confuse the issue because *C. papuanum* type is a heterotypic synonym of *A. erecta*. However, the undifferentiated stipe and blade of the specimen, and cuneate, non-zonate blade (Fig. 2f), which closely matches the gross morphological description of *A. obscura*, suggests that this specimen is not truly representative of *A. erecta* and is possibly wrongly synoymized with it. Therefore, we maintain our conclusion that the specimens recovered in Hawai‘i should be regarded as A. cf. erecta for the time being.

Avrainvillea cf. erecta was not observed at the Honolulu Harbor sites when it was resurveyed in March 2016, and reduced *Halophila decipiens* cover was found as compared to 2014. This is most likely due to scheduled dredging that occurs approximately every 15 years. Interestingly, *Halimeda kanaloana* was observed at one site where it was absent two years previously. Soft bottom assemblages in the urbanized estuary are subject to disturbance, including naturally occurring factors like storms as well as anthropogenic forces like dredging that result in light attenuation (Ruffin 1998). *Halophila decipiens* recovery from such disturbances is dependent on its seed bank when there is complete loss of its vegetative canopy (McMillan 1988). Similarly, due to its robust holdfast, it is possible that A. cf. erecta could persist below the surface of the benthos and could regrow from holdfast siphons (Hillis-Colinvaux et al. 1965, DeWreede 2006), therefore this high traffic area and benthos should continue to be monitored regularly.

The site examined near Ke‘ehi Lagoon has historically been dominated by *H. decipiens* (M. Ross, unpublished data). However, during the past two years, *H. kanaloana* has begun to appear, and in many places, is now one of the dominant species. Similarly, *Udotea* sp. was only observed for the first time in the area earlier in 2017. Based on these observations, this habitat may be undergoing significant shifts in species composition, in which A. cf. erecta is playing a part (M. Ross, unpublished data).

The morphological record for *A. erecta* (which may include genetically divergent cryptic diversity and is thus to be considered carefully) encompasses the East coast of Africa and the Red Sea to as far as the western Pacific in the waters of New Zealand and several Pacific Islands (Guiry and Guiry 2017). Given the proximity of the newly recorded O‘ahu populations to major ports and harbors, and intensity of boat traffic reaching these harbors, it is likely that the alga (e.g. as fragments) was transported here via solid ballast (Carlton 1987), sea chest, or anchor entanglement; hull fouling is an unlikely vector (unless heavily fouled to provide sufficient microhabitat) due to the normal environment and growth habit of the alga as a psammophytic species with rhizomatous holdfast.

Alternatively, the alga could have arrived as a result of Pacific currents; the Pacific Gyre carries water from Southeast Asia and Japan through the Pacific Ocean north of the Hawaiian Islands to California, and returns to the East Pacific south of the Hawaiian Islands. Additionally, the Equatorial Countercurrent feeds into the gyre, supplying it with water from Australia, New Zealand, and the Pacific Islands (Tomczak and Godfrey 1994). Introduction to Hawai‘i as a result of the 2011 Tohoku earthquake and subsequent tsunami in Japan are possible; indeed, studies have recently recorded introduced algal species on tsunami debris on the west coast of North America and in Hawai‘i (e.g. West et al. 2016, Carlton et al. 2017, Carlton et al. 2018, Hanyuda et al. 2018). Given the vegetative propagation achieved by members of the Bryopsidales, it is possible that very small fragments were carried to Hawai‘i by one means or another naturally (Hillis-Colinvaux et al. 1965, Walters and Smith 1994, Vroom et al. 2003, Wright and Davis 2006). Additionally, the close identity of DNA sequences (3-8 bp differences) obtained for the newly recorded specimens with those from Japan and Micronesia would suggest their geographical origin from the western Pacific; however, population genetic work is needed to conclusively demonstrate this connection.

Given that *A. erecta* was originally described from specimens collected from 15-36 m (Gepp and Gepp 1911), and additional records indicate that this species is also common in the intertidal to shallow subtidal (Natural History Museum 2017), this new record for the Hawaiian Islands is of serious concern, especially considering the prevalence and impacts of “*A. amadelpha*” in Hawai‘i and the “*A. amadelpha*” recently recorded in the Mediterranean Sea (Verlaque et al. 2017). “*A. amadelpha*” now inhabits the intertidal, subtidal, and mesophotic environments in Hawai‘i (Spalding 2012), and is considered invasive (Peyton 2009, Cox et al. 2017), altering the benthic ecosystem (Martinez et al. 2009), and competing with native species (Peyton 2009). Given these characteristics, considerable attention should be given to *A. erecta* in the Hawaiian Islands to monitor its possible expansion and competition with other psammophytic phototrophs in order to allow timely mitigation strategies if needed. Furthermore, these areas of high anthropogenic disturbance combined with the ecological success of this siphonous green algae makes this genus a concern for continued introductions worldwide, especially harbors experiencing heavy maritime traffic like Honolulu.

## Supplementary Material

Supplementary material 1 *Avrainvillea* sp. records and site information for the 2014 seagrass survey in Honolulu Harbor. Data type: Occurrences
File: oo_233630.xlsx Peyton KA, Foster, K

Supplementary material 2 Specimen and sequence information for those used in both morphological and molecular assessment. Data type: Specimen and sequence information.
Brief description: ARS = Sherwood Lab accession number; BISH = Bernice Pauahi Bishop Museum accession number; BM = Natural History Museum of London accession numbers TS = accession numbers of specimens provided by Thomas Sauvage; GWS = accession numbers of specimens provided by Gary Saunders.
File: oo_233631.xlsx Wade R

Supplementary material 3 Morphological characters used to identify the new *Avrainvillea* specimens and comparison with related species. Data type: Morphological
Brief description: Reference species characters retrieved from the descriptions provided by Olsen-Stojkovich (1985). Bolded character text represent character congruence with the newly discovered species from Hawai‘i.
File: oo_233632.xlsx Wade R

XML Treatment for Avrainvillea erecta

## Figures and Tables

**Figure 2a.**
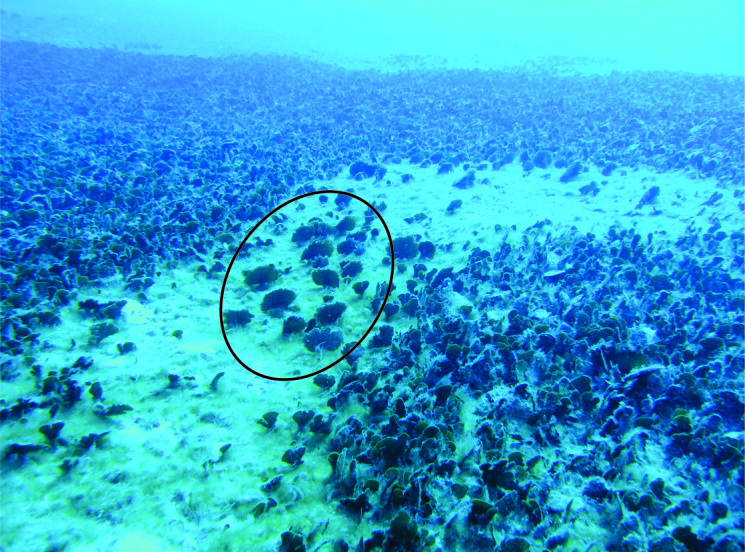
indicated by circle) within a dense bed of other siphonous autotrophs.

**Figure 2b.**
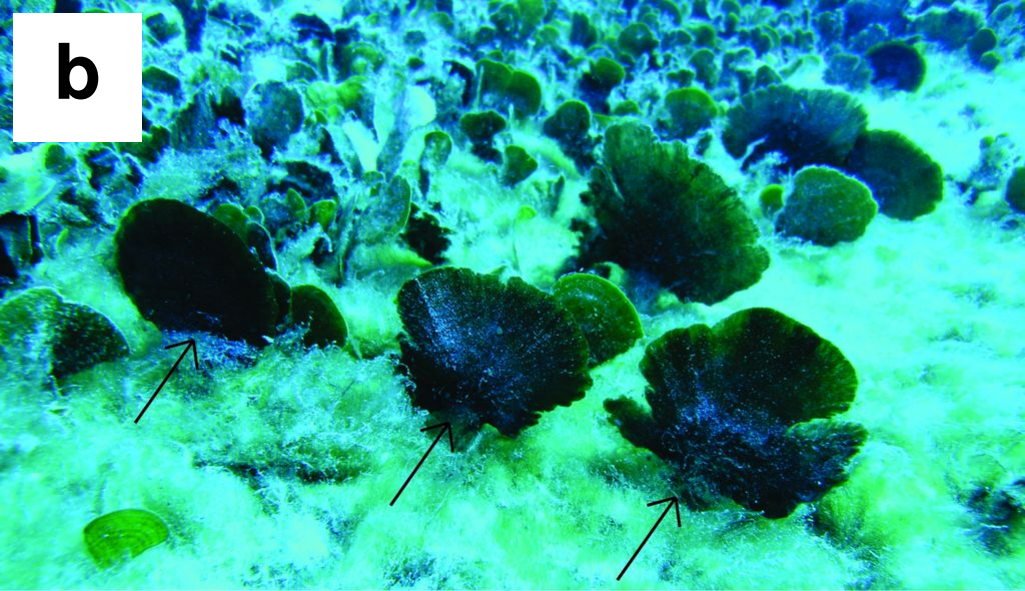
(indicated by arrow) amongst other psammophytic macroalgae, including the green algae *Udotea* sp. and *Halimeda kanaloana.*

**Figure 2c.**
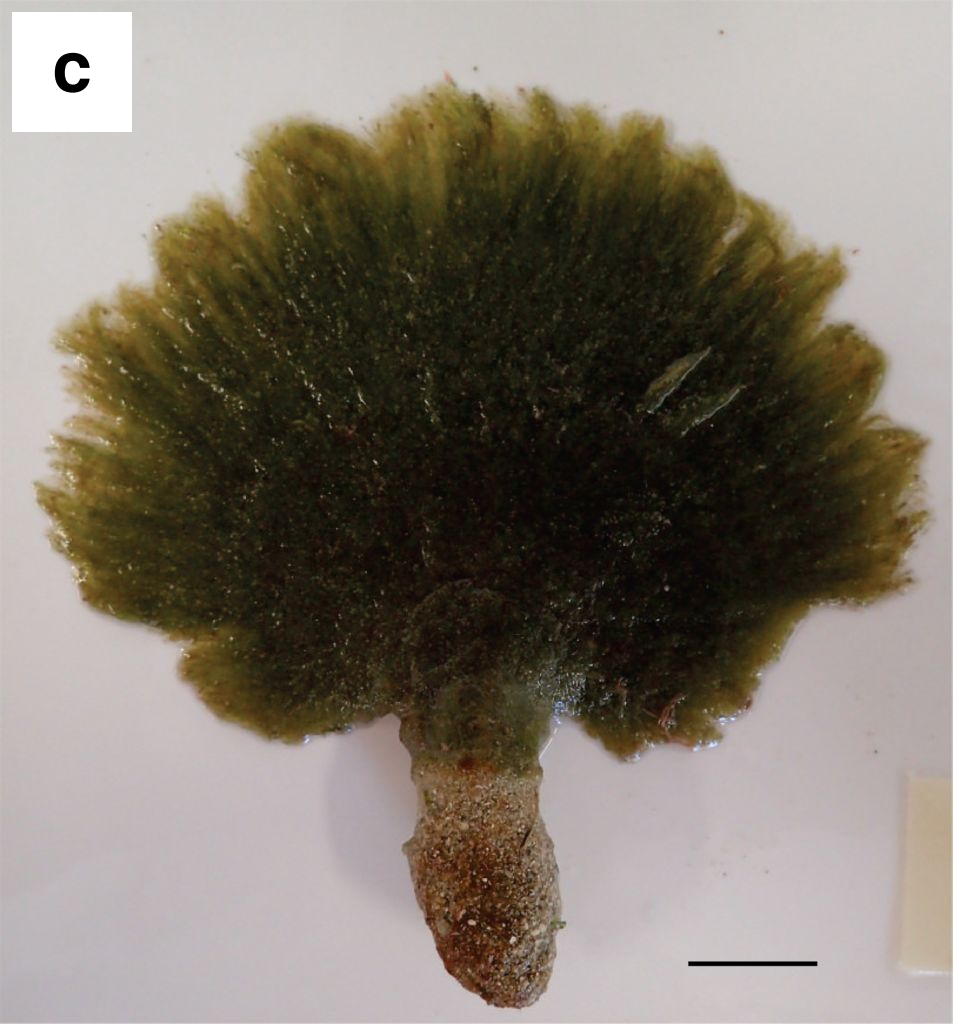
Fresh specimen of (BISH768278) exhibiting the slightly eroded margin of loose siphons, sub-reniform blade, and well-developed holdfast. Scale = 2 cm.

**Figure 2d.**
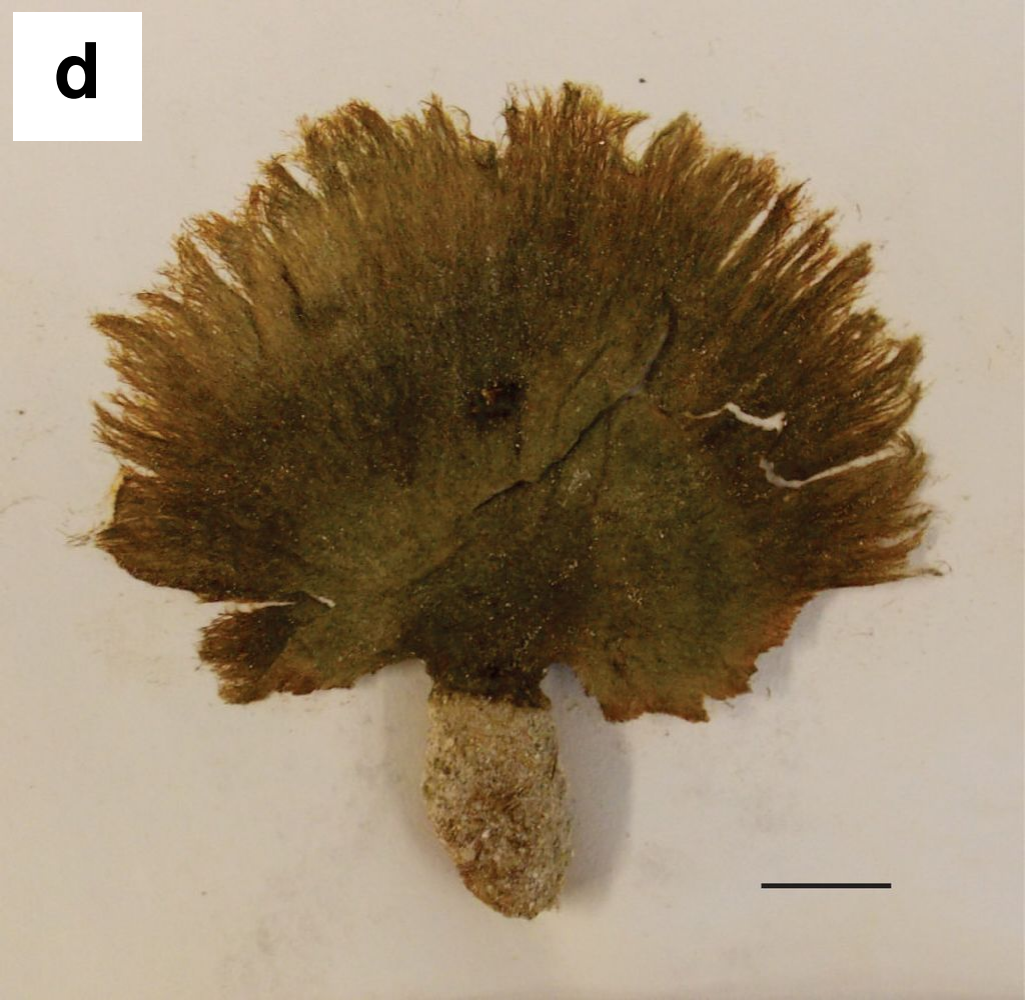
Dried specimen of (BISH768278) exhibiting the fulvous coloration and light radial zonation. Scale = 2 cm.

**Figure 2e.**
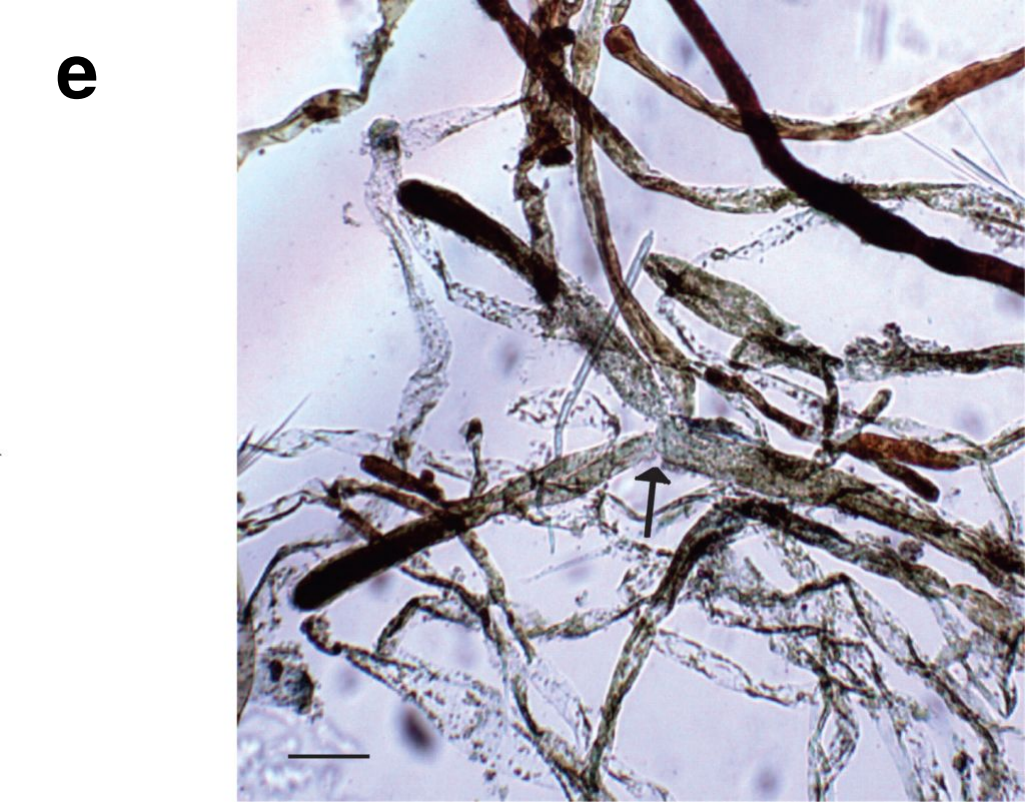
Siphons showing cylindrical to slightly torulose shape, acute constriction above the dichotomy (arrow), and fulvous coloration, particularly towards the apices. Scale = 100 micrometers.

**Figure 2f.**
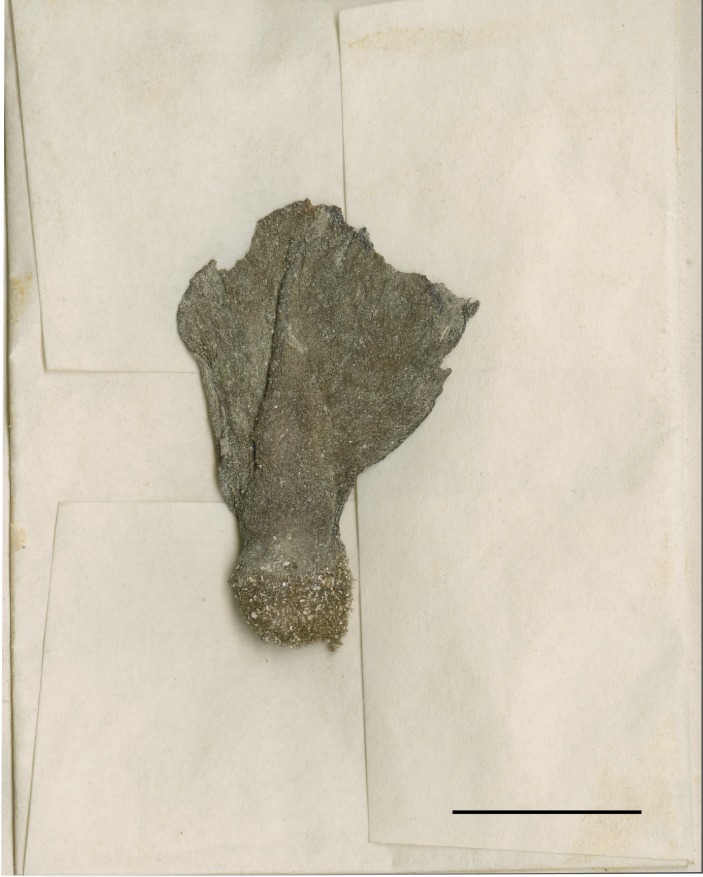
Press of the *Chloroplegma papuanum* type specimen (BM000561613), a heterotypic synonym of *Avrainvillea erecta*. Scale = 2 cm.
